# Prevention and Treatment of Postoperative Infections after Sinus Elevation Surgery: Clinical Consensus and Recommendations

**DOI:** 10.1155/2012/365809

**Published:** 2012-08-09

**Authors:** Tiziano Testori, Lorenzo Drago, Steven S. Wallace, Matteo Capelli, Fabio Galli, Francesco Zuffetti, Andrea Parenti, Matteo Deflorian, Luca Fumagalli, Roberto L. Weinstein, Carlo Maiorana, Danilo Di Stefano, Pascal Valentini, Aldo B. Giannì, Matteo Chiapasco, Raffaele Vinci, Lorenzo Pignataro, Mario Mantovani, Sara Torretta, Carlotta Pipolo, Giovanni Felisati, Giovanni Padoan, Paolo Castelnuovo, Roberto Mattina, Massimo Del Fabbro

**Affiliations:** ^1^Section of Implant Dentistry and Oral Rehabilitation, Department of Biomedical, Surgical and Dental Science, School of Dentistry, I.R.C.C.S. Galeazzi Institute, University of Milan, Via Riccardo Galeazzi 4, 20161 Milan, Italy; ^2^Laboratory of Clinical Chemistry and Microbiology, I.R.C.C.S. Galeazzi Institute, University of Milan, Milan, Italy; ^3^Department of Implantology, Columbia University, New York, NY, USA; ^4^Department of Biomedical, Surgical and Dental Science, School of Dentistry, I.R.C.C.S. Galeazzi Institute, University of Milan, Milan, Italy; ^5^Department of Oral Surgery, Dental Clinic, School of Dentistry, Istituti Clinici di Perfezionamento (ICP), University of Milan, Milan, Italy; ^6^Oral Surgery, Department of Oral Science, University Vita-San Raffaele Salute, Milan, Italy; ^7^Department of Oral Implantology, University of Corsica Pasquale Paoli, Corte, France; ^8^Department of Maxillofacial Surgery, University of Milan and Fondazione IRCCS Cà Granda, Ospedale Maggiore Policlinico, Milan, Italy; ^9^Department of Oral Surgery, School of Dental Medicine, Department of Medicine, Surgery and Dentistry, San Paolo Hospital, University of Milan, Milan, Italy; ^10^Division of Advanced Oral Surgery, Department of Dental Medicine, University Vita-Salute, San Raffaele, Milan, Italy; ^11^Otorhinolaryngology Clinic, Department of Otorhinolaryngoiatric Sciences, Fondazione IRCCS Cà Granda, Ospedale Maggiore Policlinico, Milan, Italy; ^12^Head and Neck Department, San Paolo Hospital, University of Milan, Milan, Italy; ^13^Department of Surgical Sciences, University of Insubria, Varese, Italy; ^14^Department of Public Health, Microbiology and Virology, University of Milan, Milan, Italy; ^15^Section of Oral Physiology, Department of Biomedical, Surgical and Dental Science, I.R.C.C.S. Galeazzi Institute, University of Milan, Milan, Italy

## Abstract

*Introduction*. Maxillary sinus surgery is a reliable and predictable treatment option for the prosthetic rehabilitation of the atrophic maxilla. Nevertheless, these interventions are not riskless of postoperative complications with respect to implant positioning in pristine bone. *Aim*. The aim of this paper is to report the results of a clinical consensus of experts (periodontists, implantologists, maxillofacial surgeons, ENT, and microbiology specialists) on several clinical questions and to give clinical recommendations on how to prevent, diagnose, and treat postoperative infections. *Materials and Methods*. A panel of experts in different fields of dentistry and medicine, after having reviewed the available literature on the topic and taking into account their long-standing clinical experience, gave their response to a series of clinical questions and reached a consensus. *Results and Conclusion*. The incidence of postop infections is relatively low (2%–5.6%). A multidisciplinary approach is advisable. A list of clinical recommendation are given.

## 1. Introduction

Maxillary sinus surgery can be defined as a routine and predictable procedure for the prosthetic rehabilitation in the atrophic maxilla [1–7].

In the past, implant treatment was applied to total edentulous patients [8, 9] and was later extended to partially edentulous patients; however, the resorption of the alveolar ridges in the maxilla often limits the available bone for positioning dental implants unless a reconstructive phase was performed and different classifications of bone atrophy and relative treatments protocols were proposed [10–12].

Management of patients undergoing sinus lift procedure often requires an interdisciplinary approach involving various specialists in the presurgical phase to optimize surgical results and reduce complications [13–15].

There are anatomic alterations and pathological conditions such as inflammatory-infective processes or sinus manifestations of systemic or cancer related diseases that represent contraindications and should be treated prior to maxillary sinus elevation [16, 17].

Complications are infrequent and can be easier managed if promptly diagnosed.

Postoperative infections are relatively infrequent, with infection rates reported between 2% and 5.6%, with no distinction being made between true sinus and sinus graft infections. 

Infections after sinus elevation surgery can occur in two locations. Most commonly the infection is not a true sinus infection but an infected sinus graft. It should be realized that the sinus graft is not actually in the sinus but is located below the elevated sinus membrane, hence the term subantral augmentation. True sinus infections are less common but may have more widespread consequences such as a pan-sinusitis which can occur as a result of the interconnectivity of the sinus network [18–22].

The aim of this paper is to report the results of a clinical consensus of experts (periodontists, implantologist, maxillofacial surgeons, ENT, and microbiology specialists) on several clinical questions and to give clinical recommendations on how to prevent, diagnose and treat postoperative infections.

The clinical questions addressed by the panel of experts are as follows.

What is the normal postoperative patient response to sinus surgery?
What is the correct preop and postop pharmacological treatment after sinus surgery?
In case of persistence of signs and symptoms beyond 3 weeks, what are the proper clinical recommendations?
What is the difference between early and delayed complication?
(a) Which postop infections can be managed only with pharmacological treatment? (b) Which postop infections require a combined pharmacological and surgical approach?
What are the clinical indications for a microbiologic assay?
In case of surgical management of postoperative infections, is a reentry possible and how long should the surgeon wait?
What are the most appropriate clinical recommendations to reduce the incidence of postop complications?

## 2. Materials and Methods 

A panel of experts in different fields of dentistry and medicine like periodontists, implantologists, maxillofacial surgeons, ENT, and microbiology specialists after having reviewed the available literature on the topic and taking into account their long standing clinical experience gave their response to the above mentioned questions and reached a clinical consensus.

## 3. Results

**(1) What Is the Normal Postoperative Patient Response to Sinus Surgery?**

A normal postoperative patient's response could be swelling, ecchymosis, and mild-to-moderate discomfort that is rarely spontaneous within the first few days and usually resolves within three weeks. Minor nose bleed might be present.

The resolution of symptoms after three weeks suggest a normal postop period. Usually acute spontaneous pain is absent; however, if present it is a warning sign for the clinician to investigate promptly.

**(2) What Is the Correct Preop and Postop Pharmacological Treatment after Sinus Surgery?**

Usually sinus surgery is a surgical procedure carried out under antibiotic prophylaxis and postoperative drug therapy as seen in Table 1. This pharmacological regimen is based on clinical experience and indirect evidence. In implant dentistry, there is a trend that favor the use of prophylactic antibiotics to reduce infections [23, 24].

With regard to preop or postoperative corticosteroid therapy, a common consensus was reached regarding the use of corticosteroid but not on the dosage due to the heterogeneity of the pharmacological regimens utilized by the different experts.

**(3) In Case of Persistence of Signs and Symptoms beyond 3 Weeks, What Are the Proper Clinical Recommendations?**

The presence of signs and symptoms beyond three weeks calls for a careful examination and monitoring of the patient until total recovery.

If the patient has not fully recovered after 3 weeks, CT is suggested to evaluate maxillary sinuses, nasal, and sinus endoscopy can be added if necessary.

**(4) What Is the Difference between Early and Delayed Complication?**

Early complication happens within 21 days following surgery.

Delayed complication sets in more than 21 days after the surgery.

A clear distinction between early and delayed complications allows a time-related assessment of the complication. This classification is useful in communicating among clinicians and writing scientific papers.

**(5a) Which Postop Infection Can Be Managed Only with Pharmacological Treatment?**

Graft infection well contained under the sinus membrane, as seen in the scan, with only a clean serum exudate from the surgical incision can be managed only with pharmacological treatment (Table 2).

A strict monitoring of the patient is needed until resolution of the complication.

**(5b) Which Postop Infection Require a Combined Pharmacological and Surgical Approach?**

If the graft is well contained under the schneiderian membrane (as seen in the CT scans) but signs and symptoms still persist beyond 3 weeks associated with additional symptoms (like tenderness, nasal obstruction, pain, fistulization, purulent discharge from the nose and throat, flap dehiscence, and suppuration), partial or total removal of the bone graft by oral access combined to pharmacological therapy is recommended.

If the graft is not contained under the sinus membrane and a loss of graft material inside the sinus is present (as seen in the CT scans) a multidisciplinary approach to manage the complication is mandatory. Functional endoscopic sinus surgery (FESS) could be suggested along with the removal of bone graft and dental implants from an oral approach [25].

A quick and multidisciplinary approach to the patient with sinus complications is required in these clinical scenarios.

**(6) What Are the Clinical Indications for a Microbiologic Assay?**

Microbiologic assay is always suggested but a negative result (bacteria absence) does not mean absence of infection. Usually during antibiotic therapy, bacterial cultures are negative. If possible it is recommended to make a second test some days after the end of the pharmacological therapy.

The indications to request a microbiologic assay have to be evaluated in relation to the antibiotic response in term of days versus recovery speed, seriousness of the complication, and general patient condition. A close patient monitoring is always advised.

**(7) In Case of Surgical Management of a Postoperative Infection, Is a Reentry Possible and How Long Should the Surgeon Wait?**

A sinus reentry is possible after a CT evaluation and preferably an ENT reevaluation to confirm a complete sinus healing (which on the average requires 6–9 months).

**(8) What Are the Most Appropriate Clinical Recommendations to Reduce the Incidence of Postop Complications?**

The clinical recommendation are as follows:

- careful assessment of the medical history of the patient,
- proper patient selection with healthy maxillary sinus,
- to take a pre-operative CT scan to evaluate sinus anatomy and identify preexisting pathology,
- a smoking cessation protocol is always recommended and, especially in case of heavy smokers (≥15 cigarettes a day), evaluated with caution [26],
- preventive resolution of periodontal and endodontic diseases,
- adequate antibiotic prophylaxis,
- to achieve full mouth plaque score (FMPS) and full mouth bleeding score (FMB5) <15%. In case of provisional crowns it is advisable to remove the temporary crowns and disinfect the abutments with antiseptic solution,
- preop disinfection of the skin with an antiseptic solution and mouth rinses with chlorhexidine,
- use of sterile draping and infection-control protocol,
- to keep the incision distant from the antrostomy,
- salivary-contamination prevention for bone graft and/or other biomaterials,
- intra- and postoperative control of the hemostasis,
- prevention of bone overheating,
- use of two different surgical sets of instruments: one for the flap elevation phase and the other for the grafting phase,
- to rinse the surgical field with sterile saline solution,
- to keep the surgical time as short as possible,
- postoperative chlorhexidine rinses,
- correct postoperative pharmacological therapy,
- preplanned patient controls: weekly for the first month and monthly for the following 3 months.

## 4. Conclusion

The maxillary sinus elevation procedure using a lateral window approach has been shown to be the most successful bone augmentation procedure that is performed as a pre-prosthetic procedure before implant placement [5]. When success is measured by patient outcome (success of the grafting procedure), the excellent result rate achieved is due to the fact that complications are minimal and possibly further on prevented through proper case selection, good surgical technique, and proper and prompt handling of intraoperative and postoperative complications. Properly performed sinus grafting does not alter neither sinus function [13] nor the characteristics of the voice [25]. When measured by implant outcome (implant survival rate), it has been shown that implant survival rates in the high 90th percentile can be achieved through proper decision making with regard to implant surfaces (textured), graft materials (highest survival with xenografts), and the placement of a barrier membrane over the window. Complications are infrequent and those that occur after sinus grafting procedures are for the most part localized and readily resolved. Since prevention is better than treatment, the clinical recommendations given by the panel will help in reducing the incidence of the postop infections. 

## Figures and Tables

**Table 1:** Prophylaxis and post-operative drug therapy in sinus lift patient.

	Prophylaxis	Post-operative therapy
Patient not allergic to penicillin	Amoxicillin/clavulanic acid 1 gr twice a day (BID) per os starting 24 hours before surgery	Amoxicillin/clavulanic acid 1 gr three times a day (TID) per os for 7 days
Patient allergic to penicillin	Clarithromicin 250 mg BID + Metronidazole 500 TID per os starting 24 hours before surgery	Clarithromicin 250 mg BID + Metronidazole 500 TID per os for 7 days

**Table 2:** Drug therapy for sinus lift complications.

Patient not allergic to penicillin	Amoxicillin/clavulanic acid 1 gr TID and Metronidazole 500 mg TID per os
Patient allergic to penicillin	Levofloxacin 400 mg BID per os until 72 hours to symptom remission

Usually these regimens are utilized for 7–10 days	
